# Chemical and Electrochemical Synthesis of Polypyrrole Using Carrageenan as a Dopant: Polypyrrole/Multi-Walled Carbon Nanotube Nanocomposites

**DOI:** 10.3390/polym10060632

**Published:** 2018-06-07

**Authors:** Mostafizur Rahaman, Ali Aldalbahi, Mohammed Almoiqli, Shaykha Alzahly

**Affiliations:** 1 Department of Chemistry, College of Science, King Saud University, Riyadh 11451, Saudi Arabia; 2 King Abdullah Institute for Nanotechnology, King Saud University, Riyadh 11451, Saudi Arabia; shaykha.alzahly@hotmail.com; 3 Nuclear Sciences Research Institute, King Abdulaziz City for Science and Technology, Riyadh 11442, Saudi Arabia; almoiqli@kacst.edu.sa

**Keywords:** UV-Vis spectra, electrical conductivity, morphology, mechanical property, thermal property

## Abstract

In this article, iota-carrageenan (IC) and kappa-carrageenan (KC) are used as dopants for the chemical and electrochemical synthesis of polypyrrole (PPy). The composites of chemically synthesized PPy with multi-walled carbon nanotubes (MWNTs) were prepared using an in situ technique. Both the dialyzed and non-dialyzed IC and KC were used as dopants for electrochemical polymerization of pyrrole. Chemically synthesized PPy and PPy/MWNTs composites were studied by ultraviolet visible (UV-Vis) absorption spectra to investigate the effect of the concentration and the incorporation of MWNTs. In addition, the electrical, thermal, mechanical, and microscopic characterizations of these films were performed to examine the effect of the dopants and MWNTs on these properties, along with their surface morphology. The films of electrochemically polymerized PPy were characterized using UV-Vis absorption spectra, scanning electron microscopy, and cyclic voltammetry (CV). The results were then compared with the chemical polymerized PPy.

## 1. Introduction

Conducting polymers (CPs) are organic materials that possess optical, magnetic, and electrical properties [1,2,3]. CPs can be synthesized in many different ways, such as pyrolysis, photochemical, plasma, chemical and electrochemical polymerization [3,4,5,6,7,8]. Among these methods, chemical and electrochemical polymerization methods have been most extensively used for CP synthesis. Both methods result in different properties of the CPs, e.g., different conductivity of the same polymer can be produced [9]. The chemical method is the most efficient for preparing large amounts of CPs, since it is performed without electrodes [10]. The electrochemical method of synthesis, which has high rates of accuracy and purity, is also a simple procedure to produce CPs as self-supporting and free-standing films, as well as as colloids [4,11]. The main difference between the two methods of synthesis is that the electrochemical technique produces very thin CP films, whereas the chemical procedure produces very thick films or can give CPs in the form of a powder.

Polypyrrole (PPy) is a conducting polymer with relatively high electrical conductivity and good environmental stability. This polymer is electroactive in both aqueous and organic electrolyte solutions [3]. Electrical, chemical, and mechanical properties can be controlled by switching between its oxidized and reduced states. Since PPy has been identified as non-toxic and potentially biocompatible, it has been used in a wide range of biological applications and, similarly to polyaniline, can be synthesized by chemical or electrochemical methods [3,12], leading to insolubility in common organic solvents [3]. Hence, many attempts have been made to counteract this issue, such as by using various functional dopants and polymeric co-dopants [13]. In a previous report, the chemically synthesized PPy using dodecylbenzene sulfonic acid (DBSA) as a dopant yielded conductivity of about 5 S/cm [14], whereas another study achieved 18 S/cm when doping PPy with camphor sulfonic acid (CSA) [15]. The nature of the solvent also affects the conductivity of the prepared PPy. It has been shown that electrochemically synthesized PPy doped with di(2-ethylhexyl) sulfosuccinate (DEHS) displays conductivity from 1.0 × 10^−4^ S/cm, using methyl alcohol as a solvent, and 6.6 × 10^−2^ S/cm, using oleyl alcohol [13]. Current research is finding a large range of applications for CPs, like chemical and gas sensors, corrosion inhibitors, battery electrodes, microwave shielding, drug delivery and artificial muscles [9,16,17,18,19,20].

Carrageenan is an anionic, water soluble biopolymer that is well known for its gel forming capabilities [21]. These are mainly employed in the food processing industry and for medical applications [22,23,24]. In food processing, carrageenans are used as water-based gels (such as cakes, desserts and jellies), in dairy products (such as puddings and ice-cream), and as additives to improve the texture of cooked food. In the medical field, carrageenans have been used in probiotic encapsulation, both as microcapsules and microspheres [23].

CPs can be doped with a variety of molecules, such as small salt ions [25,26], peptides [27], organic acids [28,29], polymeric acid [30,31,32] and biopolymers [33,34,35,36,37,38]. Biopolymers such as gellan gum, heparin, hyaluronic acid and chondroitin sulfate have been used to dope CPs [34,35,38]. Recently published research used the biologically derived polysaccharide, gellan gum, as a dopant for PPy [34]. In this work [34], conducting polymer electrode coatings were developed as a platform for improved functionality of neural prosthetic electrodes. Another biopolymer, heparin, has also been the subject of research as dopant for PPy [35,36,37,38]. Electrochemical analysis showed that, PPy doped with heparin had a strong affinity to the protein, thrombin, when in its oxidized state but not in its reduced state. Therefore, it finds applications in the detection and purification of thrombin [36,37].

The combination of unique properties of both CNTs and CPs has led to the production of composite materials with enhanced mechanical and electronic properties [39,40]. These composite materials can then be used in a wide variety of applications, including optical limiting devices [41], electrochemical capacitors [42] and sensors [43]. One potential use for CP/CNT composites is in photovoltaic devices and organic light emitting diodes (OLEDs) [41,44].

The main objective of this work is to check the suitability of using iota and kappa-carrageenan as dopants for the synthesis of PPy by chemical and electrochemical methods. Another reason to choose carrageenan is that it also acts as a dispersant for CNTs in chemical polymerization and as a cell electrolyte during the electrochemical polymerization process. The purpose of using CNTs was to enhance the mechanical and electrical properties of the resultant composites, as it possesses high mechanical strength and electrical conductivity. The composite materials were characterized in terms of their electrical, mechanical and other physical properties such as thermal stability and morphology. The results of chemically synthesized PPy/carrageenan composites were compared with the electrochemically synthesized ones and discussed. Finally, based on the obtained results, a potential application has been proposed for the resultant composites.

## 2. Materials and Methods

### 2.1. Materials

τ-carrageenan [(IC, molecular weight 350,000–800,000 g/mol (100–1000 kD), Genuvisco type CI-123, lot # SK93842)] and κ-carrageenan [(KC, molecular weight 350,000–800,000 g/mol (100–1000 kD), Genuvisco type CI-102, lot # SKS2500) were received as a gift from CP Kelco (Atlanta, GA, USA). Pyrrole monomer (Py, C_4_H_5_N) distilled and stored at −4 °C prior to use) and ammonium persulfate (APS, (NH_4_)_2_S_2_O_8_) were obtained from Sigma Aldrich (St. Louis, MI, USA). Methanol (CH_3_OH, lot # 318-2.5L GL) was purchased from Ajax Finechem (Taren Point, Australia).

Nylon (pore size of 0.45 μm) filtration membrane was purchased from Millipore (Burlington, MA, USA). Dialysis tubes (Spectra/Por 2 dialysis membrane, lot # 132636) were obtained from Spectrum Laboratories (Rancho Dominguez, CA, USA). Indium tin oxide coated glass slides (ITO, lot # 636916) and double-sided conductive carbon tape were purchased from Sigma Aldrich and Proscitech (Kirwan, Australia), respectively. Chemical vapor deposition produced multi-walled carbon nanotubes (MWNTs) were obtained from Nanocyl Incorporated (New York, NY, USA, lot # 090901, P0348). Milli-Q water (resistivity =18.2 MΩ·cm) from a Millipore Q water purification system was used in all experiments. All materials were used as-received.

### 2.2. Preparation of Carrageenan Solutions

Carrageenan solutions (IC and KC) were prepared to appropriate concentrations by dissolving carrageenan powder in Milli-Q water under continuously stirring for 3 h at 70 °C on a hot plate stirrer (Stuart, CB162) using a magnetic stir bar.

### 2.3. Chemical Synthesis of Polypyrrole 

The pyrrole monomer in this experiment was distilled at atmospheric pressure before starting polymerization. The PPy was chemically synthesized as follows: 12.4 μL of distilled pyrrole monomer was added to 2 mL of IC or KC (0.5% *w*/*v*) solution. The mixture was stirred at 21 °C for 2 h. 2 mL of IC or KC (0.5% *w*/*v*) solution was added to the mixture which was then stirred on a hot plate stirrer for 1 h. 20.3 mg of ammonium persulfate was dissolved in 1 mL of Milli-Q water, and then slowly added dropwise into the above mixture under stirring on a hot plate stirrer for 3 h at 21 °C.

The PPy was obtained by filtering the reaction mixture using a nylon membrane (pore size = 0.45 μm) and washing several times with Milli-Q water and methanol. Finally, the product was dried in a vacuum oven at 60 °C for 24 h.

### 2.4. Chemical Synthesis of PPy/CNT Composites

Composite of PPy/CNT was synthesized by an in situ chemical polymerization method of pyrrole in the presence of CNTs. The procedure of this method was as follows: 3 mg of CNTs were dispersed in 2 mL of IC and KC (0.5% *w*/*v*) solution by sonicating for 40 min using the digital sonicator (probe diameter =10 mm, power output = 12 W and pulse mode 0.5 s on/off). During sonication, the sample vial was placed inside an ice water bath to control the solution temperature. 12.4 μL of distilled pyrrole monomer was added to the CNT dispersion and the mixture was stirred on a hot plate stirrer at 21 °C for 2 h. 2 mL of IC or KC (0.5% *w*/*v*) solution was added to the mixture and stirred on a hot plate stirrer for 1 h. 20.3 mg of ammonium persulfate was dissolved in 1 mL of distilled water and added dropwise to the above mixture under stirring on a hot plate stirrer for 24 h at 21 °C. The PPy/CNT composites were obtained by filtering the reaction mixtures using a nylon membrane (pore size = 0.45 μm) and washing several times with Milli-Q water and methanol. The final product was dried in a vacuum oven at 60 °C for 24 h.

### 2.5. Electrochemical Synthesis of PPy

204 mg of IC or KC powder was dissolved in 40 ml of Milli-Q by stirring for about 10 min at about 45 °C. An additional set of solutions was used for dialysis by the dialysis tubing method. Standard regenerative cellulose with suitable pore size was used as a semipermeable membrane. The required solution of IC or KC was poured into dialysis tubing (Spectra/Por 2 dialysis cellulose membrane, lot # 132636, (molecular weight cut-off (MWCO) = 1 kD)) and dialyzed at 21 °C, initially, six times against 0.2 M NaCl solutions, and then six times against Milli-Q water to obtain dialyzed IC (ICd) or KC (KCd). The purpose of carrying out dialysis was to purify the polymers by removing small and unwanted compounds/impurities as the polymer macromolecules cannot pass through the semipermeable membrane. It is expected that the dialyzed polymers will exhibit better mechanical strength compared to non-dialyzed ones. After dialysis, the volume of all carrageenan solutions (IC, ICd, KC and KCd) was then increased to 50 mL by addition of Milli-Q water, to which 40 mM of pyrrole was added and dissolved by stirring for 30 min at 35 °C. The resulting solutions were then purged for 15 min with nitrogen gas in order to remove any dissolved oxygen. The temperature of all solutions was maintained between 0–5 °C using ice baths. PPy-carrageenan films were synthesized galvanostatically using a two-electrode electrochemical cell, with platinum mesh and ITO glass as auxiliary and working electrode, respectively. Current density 0.7 mA/cm^2^ was applied for 2 h in order to deposit PPy-carrageenan films onto ITO glass electrodes. The resulting coated electrodes were rinsed with Milli-Q water for 30s immediately after synthesis to remove any residual carrageenan or pyrrole monomer and then dried at 30 °C for 24 h under vacuum in oven. The resulting films (PPy-IC, PPy-ICd, PPy-KC and PPy-KCd) were peeled off the ITO glass to yield uniform free-standing films.

### 2.6. Preparation of Carrageenan-CNT Dispersions

The solutions of IC (0.8% *w*/*v*) and KC (0.5% *w*/*v*) were prepared by adding 120 and 75 mg, of IC and KC, respectively, to 15 mL of Milli-Q water while stirring on a hot plate stirrer for 3 h at 70 °C. Homogenous carrageenan-CNT dispersions were prepared by sonicating up to 50 min using the sonicator at the specified conditions mentioned earlier.

### 2.7. Preparation of Films by Evaporative Casting

Free-standing films were prepared by evaporative casting of carrageenan solutions and carrageenan-CNT dispersions. 15 mL of carrageenan solutions and carrageenan-CNT dispersions were injected into the base of cylindrical plastic containers (polystyrene, diameter = 55 mm, Lomb Scientific), which were then dried in an oven at 35 °C for 24 h. The resulting films were then peeled off the substrate to yield uniform free-standing films.

## 3. Characterization Techniques

### 3.1. UV-Vis Spectroscopy

UV-Visible-NIR absorbance spectra were obtained between 180 and 1300 nm with a UV-Vis-NIR spectrophotometer (Cary 500) using a quartz cuvette (path length = 5 mm). The stability of dispersion was measured using a UV-Vis spectrometer (Cary 50) by acquiring the absorbance at 700 nm over 24 h. Samples were diluted as required.

### 3.2. Electrical Conductivity Measurements

The electrical resistance of films was evaluated using two-point and four-point probe techniques. Current (*I*)-voltage (*V*) characteristics were determined with a two-point probe technique under controlled conditions in air (21 °C, 45% relative humidity, RH) with a waveform generator (Agilent 33220A) and a digital multimeter (Agilent 34410A).

### 3.3. Optical Microscopy

The dispersions were imaged using an optical microscope (LEICA Z16 APO) fitted with a digital camera (LEICA DFC280). The images were acquired using the Leica Application Suite (version 3.1.0 R1) (Leica Microsystems, Mumbai, India) software. Dispersions were dropped onto glass slides and printed tracks were imaged on their substrates. Images were typically acquired using 20× and 50× magnification.

### 3.4. Scanning Electron Microscope

Scanning electron microscope (SEM) images were acquired using a JEOL JSM-7500FA (Peabody, MA, USA). Samples were prepared by mounting small pieces of films onto a brass stub (11 × 5 mm^2^) using double-sided conductive carbon tape. When required, samples were coated with a thin platinum layer using an Edwards AUTO 306 Sputter system.

### 3.5. Tensile Testing

The mechanical properties of all films were obtained by using a dynamic mechanical analyzer (DMA) Q800 (TA instruments, New Castle, DE, USA). Measurements were carried out under ambient conditions (21 °C, 45% RH) on rectangular strips (length = 10 mm and width = 4–7 mm), which were placed inside a paper frame, the sides of which were cut prior to testing. All tests were carried out at a cross-head speed of 0.1 mm/min.

### 3.6. Thermogravimetric Measurements

Thermal stability measurements were carried out using a thermal gravimetric analyzer (TGA) Q500 (TA instrument, New Castle, DE, USA). An amount of ~5 mg of each sample was placed into a platinum pan and heated from 25 to 1000 °C at a heating rate of 2 °C/min and a flow rate of 90 mL/min in air.

### 3.7. Cyclic Voltammetry

Cyclic voltammograms (CV) were carried out at 21 °C using a Model 363 Princeton Applied Research Potentiostat/Galvanostat, eDAQ Model 401 E-corder and eDAQ Chart software (version 5.2.11) (eDAQ Pty Ltd, Denistone East, NSW, Australia). A triangular potential waveform was applied to the cell, cycled at a scan rate of 50 mV/s between a +300 mV upper and −700 mV lower potential at the working electrode, versus the Ag/Ag^+^ reference electrode. A current was passed between the working (conductive film) and auxiliary (platinum mesh) electrodes and the imposed potential was measured.

## 4. Results and Discussion

### 4.1. UV-Visible Spectroscopy

**Figure 1:**
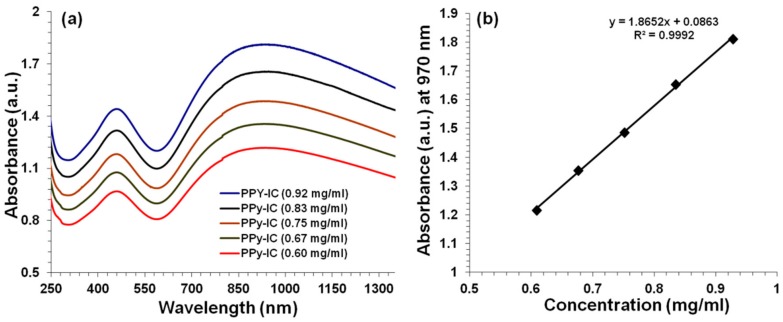
(**a**) UV-Vis absorption spectra of PPy-IC at different concentrations in Milli-Q water and (**b**) the absorbance at 970 nm of different concentrations of PPy-IC.

**Figure 2:**
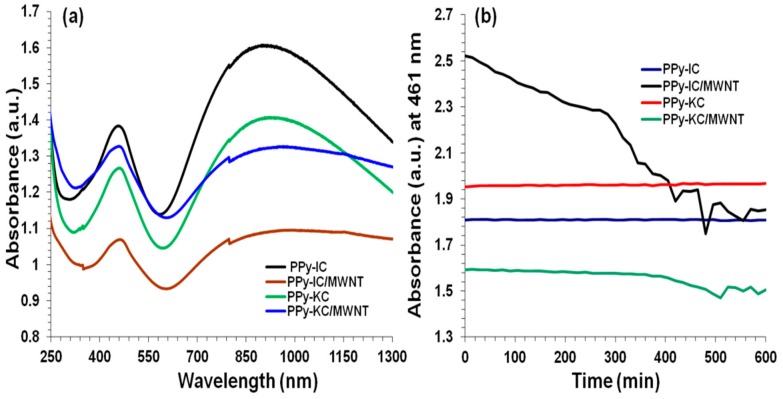
(**a**) The UV-Vis absorption of PPy-IC, PPy-KC, PPy-IC/MWNT and PPy-KC/MWNT composite solutions at a concentration of 1.0 mg/mL in Milli-Q water; (**b**) Stability of PPy-IC, PPy-KC, PPy-IC/MWNT and PPy-KC/MWNT composite solutions at a concentration of 2.0 mg/mL in Milli-Q water.

The UV-Visible absorption spectra of PPy-carrageenan and PPy-carrageenan/MWNT (3 mg MWNTs) composites solutions, synthesized by chemical polymerization method using IC and KC as dopants, have been carried out and are shown in Figure 1 and Figure 2, respectively. The effect of varying concentrations of PPy-IC composite solutions (in Milli-Q water) on the UV-Visible absorption band is presented in Figure 1a,b.

Figure 1a shows that IC doped PPy exhibits two absorption bands at 461 and 970 nm, characteristic of the PPy π–π* transition and the bipolaron band, respectively [15,45]. The absorption spectrum of PPy-IC has a long extended tail beginning at 818 nm, which indicates that PPy has adapted an expanded conformation [14,45]. Diluting the PPy-IC solution does not affect the position or the relative intensity of the absorbance bands, but decreases their intensity. For example, the intensity of the absorbance band at 461 nm of PPy-IC at 0.60 mg/mL is 31% lower compared to the intensity at 0.92 mg/mL.

Figure 1b shows that the intensity (at 970 nm) decreases linearly with decreasing PPy-IC composite concentration. The slope of this relationship is used to calculate the extinction coefficient by the Beer-Lambert equation (Equation (1)), where *A*, *C* and *l* indicate the measured absorbance, sample concentration and cuvette path length, respectively. This results in an extinction coefficient of 3.73 ± 0.03 mg/mL·cm.

(1) A=εCl,

1.0 mg/mL solutions of PPy-IC, PPy-IC/MWNT, PPy-KC and PPy-KC/MWNT were prepared in order to study the effect of incorporation of MWNTs during the polymerization process. UV-Vis spectroscopy shows that all the composite solutions exhibited two absorption bands: at 461 nm assigned as being a π–π* transition, and the bipolaron band at 970 nm (Figure 2a). These two absorption bands reveal the electron transition from valence band to anti-bipolaron band and from the valance band to the bipolaron band, respectively [14,15,45]. The effect of MWNTs is as follows. The π–π* band (461 nm) is red shifted and broadened which has been previously attributed to absorption of polymer onto the CNT surface that is facilitated by polymer backbone exposure [46,47]. In addition, the bipolaron band (970 nm) is broadened, which is indicative of a change in the conjugation length [15,48].

Figure 2b shows that the PPy-carrageenan solutions were found to be stable for at least 10 h whereas, incorporation of MWNTs into PPy-IC results in an instant decrease in the UV-Vis intensity at 461 nm. In contrast, the PPy-KC/MWNT dispersion exhibits a constant intensity for up to 5 12 h. The decrease in intensity is an indication of the unstable nature of the dispersion as it represents the settling out of materials from solution.

It is necessary to point out that the solubility of chemically and electrochemically synthesized PPy can, in general, be quite poor. It is almost insoluble in water and all common organic solvents, which in turn restricts its processability. Previous attempts have been made to improve the solubility of PPy by designing colloidal forms using the protonation and surfactant with an organic acid [14,49,50]. In this work, carrageenan has been used as a dopant to improve the solubility of PPy and, hence, its processability. A solubility test was performed using different solvents. The composite solution of PPy-IC at a concentration of 2 mg/mL was dissolved in: Milli-Q water and dimethyl sulfoxide (DMSO) (Figure S1, see Supplementary Section,). It can be seen that, the PPy-IC composite solution was soluble in Milli-Q water and DMSO solvents.

PPy films, synthesized using electrochemical polymerization of pyrrole with IC and KC biopolymers (PPy-IC and PPy-KC) were treated with dialysis against NaCl (PPy-ICd and PPy-KCd) in order to enhance the purity of these commercial products. During this process, the deposition of the films, onto ITO glass electrodes, (Figure S2, see Supplementary Section) was carried out by galvanostatic techniques.

**Figure 3:**
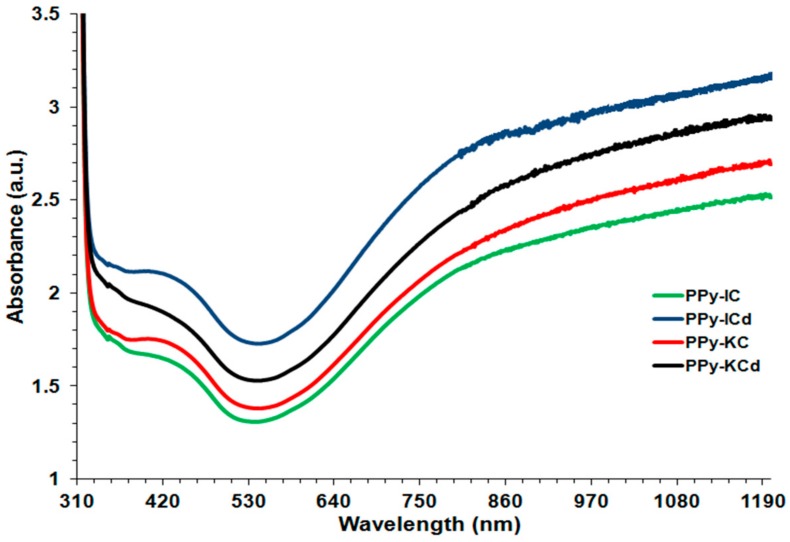
UV-Vis absorption spectra of PPy-carrageenan films synthesized using IC and KC treated with and without dialysis against NaCl.

UV-Vis absorption spectra (Figure 3) for transparent PPy films synthesized using IC and KC were analyzed in order to verify that PPy was obtained. Polymerization was allowed to proceed for just 5 min to avoid thick, opaque films. All films showed the characteristic absorption bands at 450 and 980 nm, assigned as π–π* absorption and the bipolaron bands, respectively [45,51]. These were in agreement with the bands observed for PPy prepared by chemical polymerization as mentioned earlier. It can be seen that dialysis treatment of the IC and KC results in an increase in the intensity of absorption bands of the PPy-carrageenan films. For example, at 450 nm, the absorption of PPy-IC was 1.6 au, compared to 2.1 au PPy-ICd (Figure 3). This shows that there is an increase in the purity of the carrageenan dopants due to the dialysis treatment.

### 4.2. Microscopy of Composite Films

**Figure 4:**
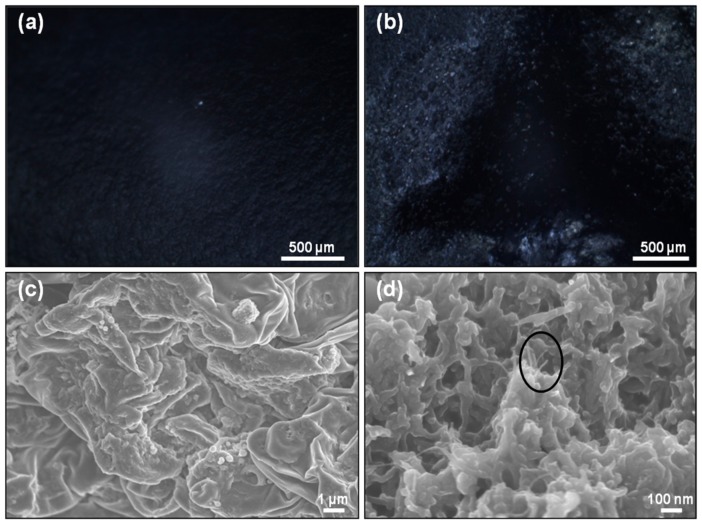
Optical microscope and SEM images of: (**a**,**c**) PPy-IC and (**b**,**d**) PPy-IC/MWNT composite films, respectively. Circle indicates the presence of MWNTs.

The free-standing films of PPy-IC and PPy-KC solutions, and PPy-IC/MWNT and PPy-KC/MWNT dispersions were prepared by evaporative casting (Figure S3, see Supplementary Section). The surface morphology was investigated using optical and electron microscopy (Figure 4).

By comparing the surface morphology of PPy-IC with and without MWNTs, it can be seen in Figure 4a,b that the presence of MWNTs resulted in a rougher film surface. SEM images revealed that the PPy-IC contains nodules with a diameter of 200–400 nm that are embedded in the IC matrix (Figure 4c). In contrast, PPy-IC films with MWNT showed a tubular morphology (Figure 4d). In addition, MWNTs are clearly identifiable and protrude from the matrix. The diameter of these MWNTs (50–60 nm) is consistent with polymer covered MWNT. This surface non-homogenous morphology is characteristic of this polymerization method which tends to form films with some aggregates compared to those prepared using electrochemical polymerization that produces a homogeneous surface morphology [52].

**Figure 5:**
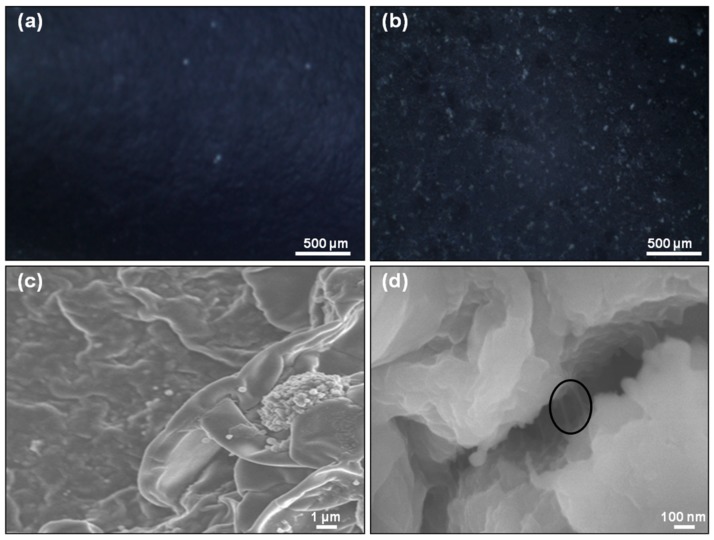
Optical microscope and SEM images of (**a**,**c**) PPy-KC and (**b**,**d**) PPy-KC/MWNT composite films, respectively. Circle indicates the presence of MWNTs.

Similar surface morphology was observed for PPy doped with KC and its composites with MWNTs (Figure 5a–d). As with IC doped PPy, the incorporation of MWNTs changed the morphology from nodular to tubular; however, PPy-KC/MWNT (Figure 5d) appears to have more MWNT coverage compared to PPy-IC/MWNT.

**Figure 6:**
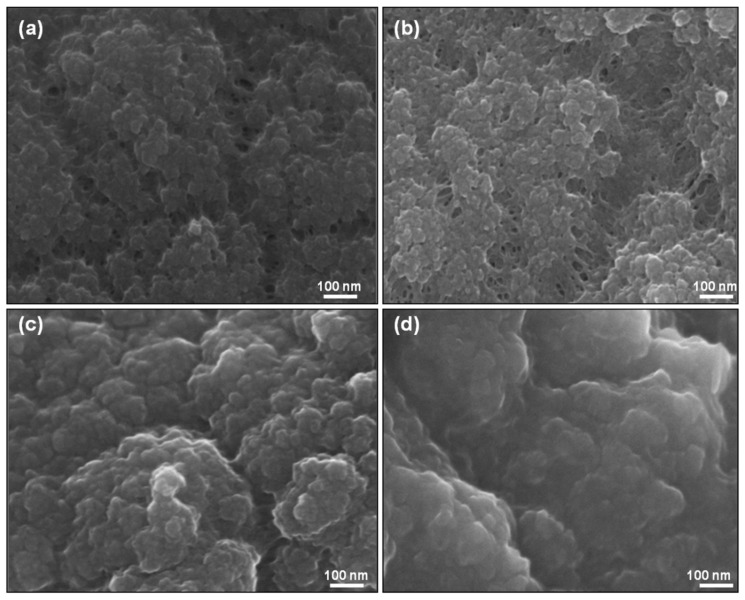
SEM images of PPy films synthesized using: (**a**,**b**) IC treated without and with dialysis, respectively and (**c**,**d**) KC treated without and with dialysis, respectively.

The SEM images of electropolymerized PPy-IC and PPy-KC films without and with dialysis are presented in Figure 6. It is revealed that all films exhibited a smooth surface, which is expected for this electropolymerization method, as discussed earlier. SEM images reveal that PPy-carrageenan contains nodular morphology with diameter 200–600 nm and is embedded in the IC and KC matrices. This nodular morphology is commonly associated with electropolymerized PPy films [3,33]. Similar results were also observed for PPy doped with other polysaccharides, such as hyaluronic acid and gellan gum [33,34].

The surface morphology is well known to be greatly affected by the type of dopant that is used during polymerization [3]. Interestingly, PPy-IC films have a porous surface structure compared to the non-porous structure which is observed for PPy-KC films (Figure 6b,d, respectively). This could be attributed to the difference in their viscosity. The measured viscosity (at shear rate 21 s^−1^ and concentration 0.5 *w*/*v*) of IC and KC are 30 ± 2 and 124 ± 4, respectively. Furthermore, PPy spherical particles are visible in the films prepared without dialyzing the KC, but almost entirely covered by biopolymer in the film prepared with dialysis (Figure 6c,d, respectively).

### 4.3. Electrical Conductivity of Composite Films

**Figure 7:**
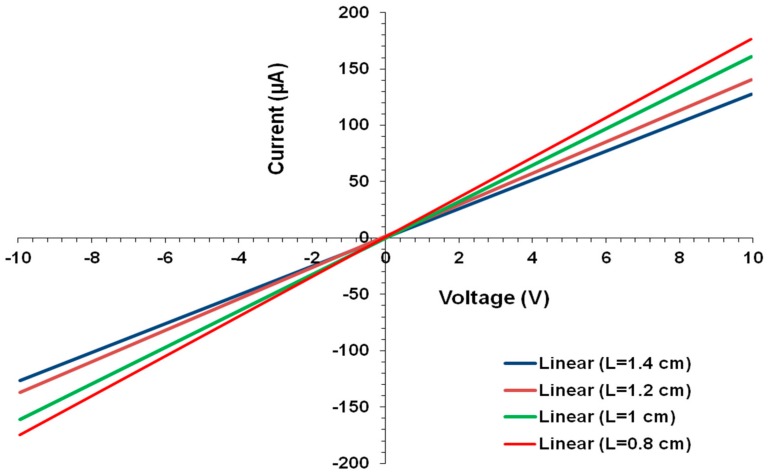
Typical *I-V* plots obtained for PPy-KC/MWNT composite films as a function of sample length (*l*).

**Figure 8:**
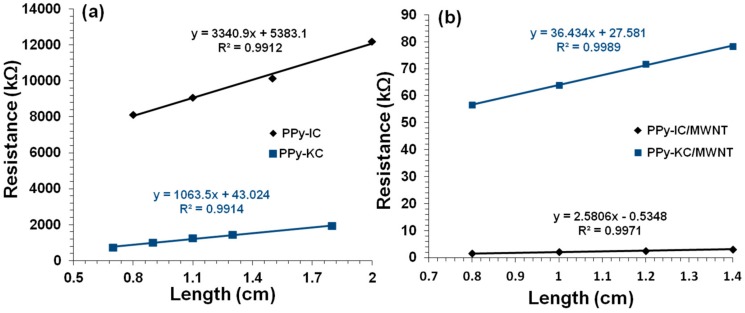
Resistance versus length for (**a**) PPy-IC (diamonds) and PPy-KC (squares) composite films and (**b**) PPy-IC/MWNT (diamonds) and PPy-KC/MWNT (squares) composite films.

The current-voltage (*I-V*) characteristics of all composite films are linear, which indicate Ohmic behavior (Figure 7). The resistance is found to increase linearly with sample length as is evident from Figure 8. The conductivity is then calculated from the slope of the straight line fit and the sample cross-sectional area.

The PPy-IC composite film exhibited lower conductivity values compared to those observed for PPy-KC (Table 1). This could be due to the higher degree of PPy polymer surface coating of IC, hence reducing the number of electrical junctions. The conductivity results achieved in this research were lower than those of low molecular weight dopants; this may be attributed to the larger carrageenan molecules wrapping around the PPy, which would reduce the conducting pathways resulting in a lower conductivity. Studies that used low molecular weight dopants reported conductivities of 50 S/m, 200 S/m and 300 S/cm [13,14,45].

**Figure 9:**
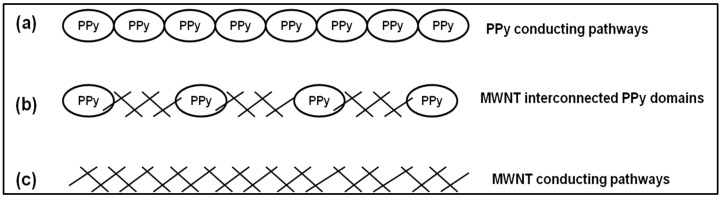
Schematic of three possible conducting pathways for electricity in PPy-carrageenan/MWNT composites.

Table 1 shows that incorporation of MWNTs significantly enhances the conductivity of the PPy-carrageenan films. For example, the conductivity of PPy-IC increases from 0.022 ± 0.004 to 21 ± 3 S/m. Similarly, the conductivity of PPy-KC was enhanced by two orders of magnitude through addition of MWNTs. These order of magnitude increases can be attributed to MWNT interconnecting some of the PPy conducting domains [53,54]. This effect may be explained as follows: there are three possible conducting pathways (Figure 9a–c). These are: overlapping PPy conducting domains, PPy domains that are interconnected by MWNTs and percolating MWNT pathways. In the absence of CNTs, only the overlapping PPy conducting pathway is possible for electrical percolation, by incorporation of CNTs all three conduction mechanisms are possible and hence, the conductivity is increased. SEM images (Figure 4 and Figure 5) show some evidence that MWNTs are interconnecting the PPy domains, which could be used to explain the increase in conductivity (Table 1) by incorporation of CNTs into the polymer matrix.

Previous studies have reported a conductivity increase from 1.56 to 3.89 S/cm with the addition of 3 wt % MWNTs to PPy doped with the cationic surfactant cetyltrimethylammonium bromide (CTAB) [55], where other research has reported increases of one and two orders of magnitude when PPy-CNT was doped with HCl and CTAB, respectively [56]. This suggests that the nature of the dopant plays an important role in the conductivity of PPy composite films.

The electrical conductivity of PPy-carrageenan films synthesized electrochemically is reported in Table 2. The results indicate that dialysis treatment of the carrageenan reduces the conductivity by 27% of the PPy-carrageenan films. For example, the conductivity of the film prepared PPy-KC film was 631 ± 66 compared to 463 ± 50 S/m of the PPy-KCd film. This difference in conductivity could be attributed to coverage of PPy by the biopolymer as observed by SEM results (Figure 6). The insulating biopolymer may act as a barrier to electrical transport. By comparing this conductivity data to what was measured for chemically polymerized PPy, the conductivity was 5 orders of magnitude higher. It has been reported that the method of synthesis, the type of dopants, the solvent and the monomer concentration influence the conductivity of the PPy [3]. In addition, in general, the electrical conductivity of chemically prepared PPy is lower than that of PPy films prepared electrochemically [57].

### 4.4. Cyclic Voltammetry of PPy Films

**Figure 10:**
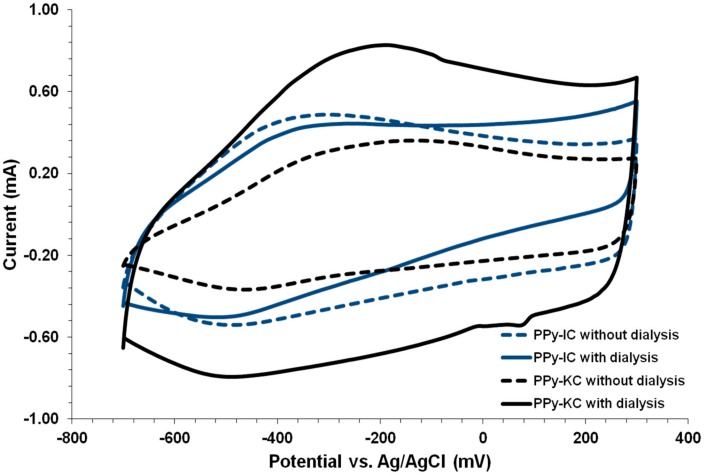
Cyclic voltammograms (in 0.1 M HCl) of PPy-carrageenan films synthesized using IC and KC treated with and without dialysis against NaCl.

The CV study was carried out only for electrochemically synthesized PPy films and their blends with IC and KC. Similar to PANI, PPy is also able to undergo oxidative and reductive chemical processes [3]. The CV spectra of PPy films synthesized using IC and KC treated with and without dialysis is shown in Figure 10. All films exhibited the characteristic PPy redox couples. The doping with IC results in oxidation and reduction peaks at −350 and −490 mV, respectively (Figure 10). These results are in good agreement with those observed for PPy doped with the polysaccharide gellan gum, which exhibited oxidation and reduction peaks at −330 and −470 mV, respectively [34]. In contrast, doping with KC resulted in oxidation and reduction peaks at −250 and −490 mV, respectively. The dialysis treatment served to shift the reduction peaks of PPy-IC and PPy-KC to a higher potential (from −490 to −515 mV). In addition, the oxidation peak for PPy-KC was shifted to a higher potential whereas, PPy-IC was not. Hence, the CV is influenced by the type of carrageenan as well as dialysis treatment.

### 4.5. Mechanical Properties of Composite Films

**Figure 11:**
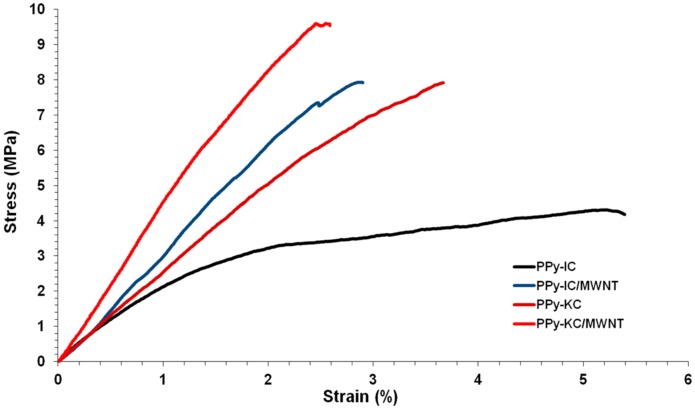
Stress-strain curves for PPy-IC, PPy-KC, PPy-IC/MWNT and PPy-KC/MWNT composite films.

The stress-strain behavior of chemically synthesized PPy composites is shown in Figure 11, and their extracted tensile properties are shown in Table 3. The figure shows that PPy films doped with IC are more flexible than the KC-produced films. For example, the elongation at break (γ) value of the PPy-IC composite film was 66% higher compared to PPy-KC composite film (Table 3). However, the PPy-KC composite film exhibited higher tensile strength (TS) and modulus (*E*) values than the PPy-IC composite film. For instance, the TS value of PPy-KC composite film is 47% higher compared to the PPy-IC composite film. This suggests that the PPy-KC composite film is stiffer and stronger, but less ductile than PPy-IC composite film.

Incorporation of MWNTs resulted in a reinforcement effect, i.e., increases in the TS and *E* values, compared to those of PPy-carrageenan films. For example, the TS value of PPy-IC composite films increases from 4.18 to 7.95 MPa (Table 3). In addition, a noticeable decrease in the γ value is observed after adding MWNTs to the composite films. These observations can be attributed to the well-known mechanical reinforcement effect of CNTs to a polymer matrix which comes at a cost in ductility [40,58,59].

By comparing these mechanical properties of PPy films doped with carrageenan to previously studied PPy doped with dodecylbenzene sulfonic acid (DBSA) [14], it was found that the latter one was six times more flexible and ductile (higher strain-at-break value) than the former one. These discrepancies may be attributed to the nature and molecular structure of the dopant.

**Figure 12:**
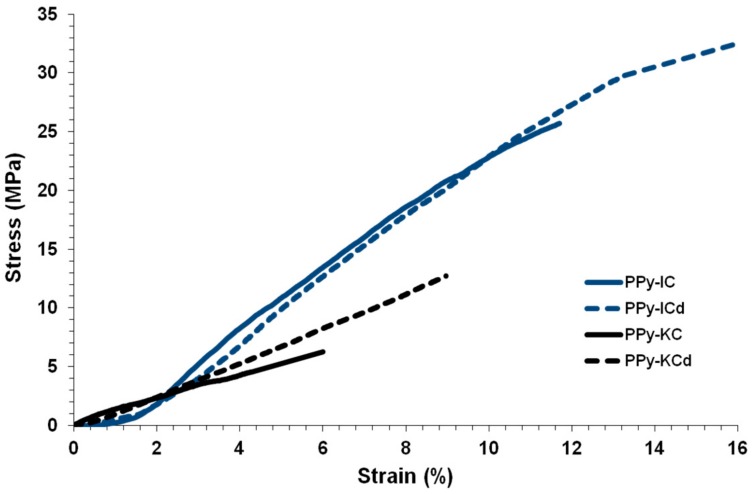
Stress-strain curves of PPy-carrageenan films synthesized using IC and KC treated with and without dialysis.

The mechanical properties of PPy films synthesized electrochemically using IC and KC treated with and without dialysis is presented in Figure 12, and the extracted tensile parameters are shown in Table 4. Tensile testing results show that PPy-IC films are stiffer, stronger, tougher and more ductile compared to PPy-KC films. Dialyzing both IC and KC prior to film formation had an additional strengthening effect. For example, without dialysis of IC, the γ and TS values of the PPy-IC films were 63% and 37% higher, respectively, compared to the PPy-ICd film. This increase in mechanical properties could be attributed to the higher degree of biopolymer surface coverage due to the dialysis treatment which was observed in Figure 6. These results confirm that the mechanical properties of PPy films are influenced by the type of carrageenan as well as dialysis treatment.

By comparing these mechanical properties with those measured for PPy films prepared using chemical polymerization, it can be seen that the TS, γ, *T* and *E* values of electrochemically synthesized PPy-IC were all higher than for chemically synthesized PPy-IC films. However, electrochemically synthesized PPy-KC only exhibited an increase in γ and T values. Thus, it is evident that the synthesis method impacts the mechanical properties of PPy films [60].

### 4.6. Thermal Stability of Composite Films

**Figure 13:**
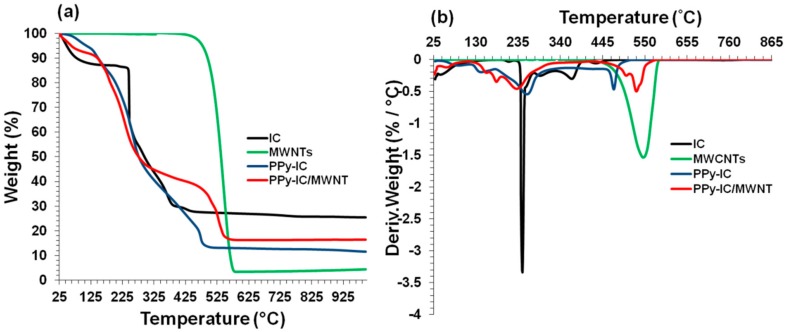
Thermogravimetric analysis (weight loss and derivative) of (**a**,**b**) IC, MWNTs, PPy-IC and PPy-IC/MWNT composite films synthesized by the chemical polymerization method using IC as a dopant.

**Figure 14:**
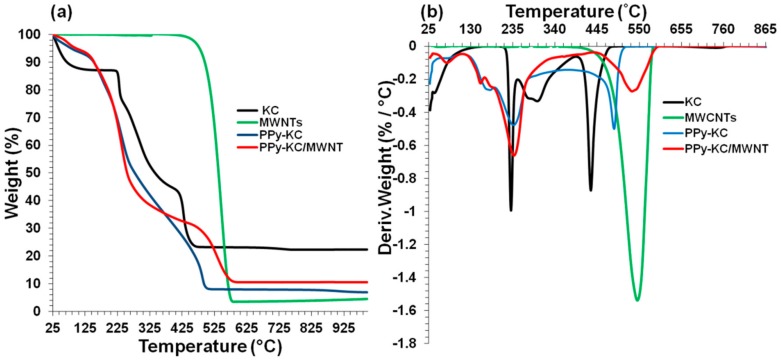
Thermogravimetric analysis (weight loss and derivative) of (**a**,**b**) KC, MWNTs, PPy-KC and PPy-KC/MWNT composite films synthesized by the chemical polymerization method using IC as a dopant.

The TG and DTG curves of PPy-IC and PPy-KC composite films are shown in Figure 13 and Figure 14. The IC film in the TGA curve exhibits a weight loss in two stages; the first stage ranged between 25 and 105 °C, with a weight loss of about 13% which corresponds to the evaporation of water. The second stage occurred in the temperature range between 200 and 480 °C and exhibited DTG peaks at 246 and 368 °C (Figure 13b), which are related to the decomposition of the IC chain structure. Figure 13a shows that only 25% mass remained at 800 °C, in agreement with literature reports [59,61]. The peak at 265 °C (Figure 13b) is attributed to the presence of IC biopolymer whereas the peak at 475 °C is due to the presence of PPy and only 12% mass of the PPy-IC remained at 800 °C.

From Figure 13a,b, it can be seen that MWNTs were comparatively more stable and did not show dramatic decomposition in the temperature range of 25–400 °C. An unsteady mass decrease was found (between 400–570 °C) due to the decomposition of MWNTs which exhibited a DTG peak at 546 °C and only 4% mass remained at 800 °C.

The trend of the TGA curve of PPy-IC/MWNT is similar to that of the PPy-IC composite films. However, the decomposition of PPy shifted to a higher temperature (503 °C). This indicates that the decomposition of the PPy-IC/MWNT composite film is mainly controlled by PPy [53]. PPy-IC and PPy-IC/MWNT composite films exhibit mass loss (6%) in the temperature range between 25–120 °C due to the evaporation of water. It is known that PPy is hygroscopic and during the heating to 120 °C the residual water evaporates [53]. At 800 °C, 16% of the PPy-IC/MWNT materials remained.

Similar results were obtained for composite films with KC (Figure 14). However, unlike PPy-IC/MWNT, the PPy and MWNT characteristic DTG peaks in PPy-KC/MWNT combined into a single peak at 530 °C. This result suggested that incorporation of MWNTs served to improve the thermal stability of the composite films.

**Figure 15:**
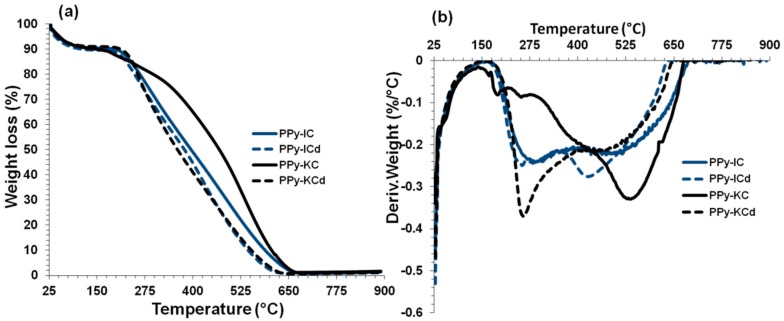
Thermogravimetric analysis (weight loss and derivative) of (**a**,**b**) PPy-carrageenan films synthesized using IC and KC treated with and without dialysis.

TG and DTG curves of electrochemically synthesized PPy films using IC and KC treated with and without dialysis are shown in Figure 15a,b. The PPy films exhibited mass loss in the temperature range between 25–120 °C, and showed only 9% mass loss which corresponds to the evaporation of water [53]. The main mass loss of PPy-carrageenan films occurred in the range of 200–626 °C for PPy films with dialyzed carrageenans and in the range of 200–666 °C for PPy films without dialyzing the carrageenans. IC and KC display a characteristic peak at ~250 °C, which was shown earlier. The derivative weight-loss curves exhibit two peaks within this range at 250 and 525 °C (Figure 15b), which correspond to carrageenan and PPy polymer degradation, respectively. Dialysis of carrageenan prior to the electrochemical synthesis of PPy-carrageenan films results in an accelerated degradation of the PPy due to the increased quantity of carrageenan, which decomposes at a lower temperature. For example, the dialysis treatment of PPy-IC film shifted the decomposition temperature from 510 to 432 °C, whereas the PPy-KC film showed a shift from 544 to 514 °C. Hence, the PPy films prepared without dialyzing the carrageenans resulted in an improved thermal stability compared to those prepared with dialysis.

Contrary to the PPy films produced by chemical polymerization using IC and KC as a dopant, which began to decompose just after 120 °C, using electrochemical polymerization method resulted in an improved thermal stability of PPy films, which did not decompose until 200 °C. Therefore, it is clear that the thermal stability of PPy films is influenced by the polymerization method.

To compare the thermal degradation/stability behavior of the films, the temperature at 5%, 20%, and 50% weight loss were considered and the extracted parameters from the above Figure 13, Figure 14 and Figure 15 are shown in Table 5. MWNTs showed the highest temperature at any weight loss because of its higher thermal stability. Although the thermal stability at 5% and 20% weight loss for IC were higher compared to KC, for 50% weight loss, the opposite trend was observed. PPy-IC and PPy-KC, synthesized chemically, showed higher thermal stability at 5% weight loss but lower thermal stability at 20% and 50% weight loss compared to their electrochemically synthesized counterparts. It was observed that the dialyzed films (PPy-ICd and PPy-KCd) showed less thermal stability value at all the weight loss percent compared to non-dialyzed ones (PPy-IC and PPy-KC). MWNTs filled composites exhibit different trend of their thermal behavior. For PPy-IC filled MWNT composite, there were high thermal stability values at 5% and 20% weight loss, but a lower thermal stability value at 50% weight loss compared to PPy-IC one. An opposite trend was observed for PPy-KC filled MWNT composite; that is, there were lower thermal stability values at 5% and 20% weight loss, but a high thermal stability value at 50% weight loss compared to PPy-KC. Hence, it can be inferred that the thermal behavior of the films does not follow any proper order. The discrepancy in the observed results may be attributed to the multiple degradation stages of the polymers and their level of interaction with MWNTs.

### 4.7. Incorporation of MWNTs

It was attempted to disperse MWNTs into the biopolymers; however, once the electrochemical polymerization was preformed, the MWNTs did not deposit onto the substrate along with PPy. This could be due to the neutral charge carried by CNTs and may be overcome by using functionalized CNTs. The charge on the nanotubes both stabilizes the dispersion and allows them to act as the counter ion or dopant for the electropolymerization of the monomer. Subsequently, the nanotubes are, to some degree, ionically bound to the polymer. Therefore, mixtures of nanotube and acid-anion doped PPy might be anticipated in the composite deposit [62]. Hence the composite films containing CNTs were not achieved.

## 5. Conclusions

The UV-Vis absorption spectrum of PPy-IC at 461 and 970 nm assigned as π–π* transition and the bipolaron band, respectively. The absorption band at 461 nm in PPy-IC is red shifted and broadened in the presence of MWNTs. The PPy-IC composite films exhibited lower conductivity (0.02 S/m), lower mechanical stiffness (4.2 MPa) but higher ductility (5.4%) compared to PPy-KC composite (0.07 S/m, 7.8 MPa and 3.6%, respectively) when synthesized chemically. Incorporation of MWNTs resulted in rough surface and tubular morphology, mechanical reinforcement at the cost in ductility and improvement of the thermal stability. In addition, it was found that the conductivity was increased by 3 and 2 orders of magnitude when MWNTs were added to PPy-IC and PPy-KC, respectively. The UV-Vis spectroscopy exhibited increases in absorbance for electrochemically synthesized PPy-IC and PPy-KC that were dialyzed; hence, this suggests that the dialysis treatment purified the biopolymers. It was found that using IC as a dopant resulted in lower electrical conductivity but higher mechanical properties compared to using KC as a dopant. It was also noted that PPy-IC film surfaces had a porous morphology, whereas PPy-KC films exhibited non-porous surfaces. This could be attributed to the difference in the viscosity between the two types of carrageenan biopolymer. The conductivity of the PPy-carrageenan films was reduced by 27% compared to undialyzed films; however, the dialysis resulted in an improvement in the mechanical properties. Morphological study showed that the surface coverage was greatly increased, while the thermal stability was reduced due to dialysis treatment.

From the above discussion and the demonstrated results, it is evident that the biopolymer carrageenan can be used as a dopant in the synthesis of conducting electroactive PPy materials with suitable electrical and mechanical properties. The results of electrical conductivity fall within the semiconductor/conductor range, indicating that these composites can be used for electronic application especially as electromagnetic interference shielding material. The porous composite can be suitable for electrode material in capacitive deionization cell.

## Figures and Tables

**Table 1:** Conductivity of casted films of PPy-IC, PPy-KC, PPy-IC/MWNT and PPy-KC/MWNT composite solutions.

Sample	σ (S/m)
PPy-IC	0.022 ± 0.004
PPy-IC/MWNT	21 ± 3
PPy-KC	0.067 ± 0.012
PPy-KC/MWNT	2.2 ± 0.4

**Table 2:** Conductivity of PPy-carrageenan films synthesized using IC and KC treated with and without dialysis against NaCl.

Film	Thickness (cm)	Rs (Ω·m)	σ (S/m)
PPy-IC	0.0045 ± 0.002	35.2 ± 3.0	631 ± 66
PPy-ICd	0.0011 ± 0.001	183 ± 3.2	463 ± 50
PPy-KC	0.0034 ± 0.002	43.8 ± 2.7	674 ± 49
PPy-KCd	0.0021 ± 0.001	93.2 ± 2.9	501 ± 31

**Table 3:** Mechanical properties of PPy-IC, PPy-KC, PPy-IC/MWNT and PPy-KC/MWNT composite films, prepared by evaporative casting of their composite solutions: Tensile strength (TS), strain-at-break (γ), toughness (*T*) and Young’s modulus (*E*).

Film	TS (MPa)	γ (%)	*T* (J/m^3^)	*E* (MPa)
PPy-IC	4.18 ± 0.97	5.39 ± 0.43	0.17 ± 0.02	232 ± 6
PPy-IC/MWCNT	7.95 ± 1.16	2.93 ± 0.23	0.13 ± 0.01	361 ± 9
PPy-KC	7.83 ± 1.12	3.58 ± 0.35	0.16 ± 0.02	257 ± 7
PPy-KC/MWCNT	9.54 ± 1.31	2.51 ± 0.21	0.14 ± 0.01	453 ± 12

**Table 4:** Mechanical properties of PPy-carrageenan films prepared by using IC and KC treated with and without dialysis against NaCl: Tensile strength (TS), strain-at-break (γ), toughness (*T*) and Young’s modulus (*E*).

Film	TS (MPa)	γ (%)	*T* (J/m^3^)	*E* (MPa)
PPy-IC	25.2 ± 3.6	11.6 ± 2.3	1.3 ± 0.1	256 ± 13
PPy-ICd	34.6 ± 6.8	18.9 ± 6.1	1.8 ± 0.3	282 ± 16
PPy-KC	6.5 ± 3.1	6.7 ± 1.1	0.2 ± 0.1	186 ± 14
PPy-KCd	12.6 ± 5.6	8.9 ± 3.7	0.5 ± 0.2	199 ± 13

**Table 5:** Temperature at different percent weight loss.

Films	Temperature at 5% weight loss (°C)	Temperature at 20% weight loss (°C)	Temperature at 50% weight loss (°C)
MWNTs	479.0	512.0	573.0
IC	048.2	246.0	295.0
* PPy-IC	062.9	186.0	376.0
# PPy-IC	047.6	262.0	395.0
# PPy-ICd	040.8	248.0	378.0
* PPy-IC/MWNT	111.2	203.0	277.0
KC	043.5	232.0	341.0
* PPy-KC	098.0	196.0	255.0
# PPy-KC	051.7	295.0	469.0
# PPy-KCd	046.7	256.0	360.0
* PPy-KC/MWNT	081.7	192.0	278.0

* Chemically synthesized, # electrochemically synthesized.

## Supplementary Materials

The following are available online at http://www.mdpi.com/2073-4360/10/6/632/s1.
