# Supplementary material for: Chemical and Electrochemical Synthesis of Polypyrrole Using Carrageenan as a Dopant: Polypyrrole/Multi-Walled Carbon Nanotube Nanocomposites
Source: Polymers (Basel). 2018 Jun 7;10(6):632. doi: 10.3390/polym10060632 (PMC6403922; doi:10.3390/polym10060632)
Supplement: Supplementary file 1 [file polymers-10-00632-s001.pdf]

polymers

MDPI

Supplementary Information

Chemical and Electrochemical Synthesis of Polypyrrole Using Carrageenan as a Dopant: Polypyrrole/Multi-Walled Carbon Nanotube Nanocomposites

Mostafizur Rahaman [1,*] Ali Aldalbahi [1,2,*], Mohammed Almoiqli [3] and Shaykha Alzahly [2]

[1] Department of Chemistry, College of Science, King Saud University, Riyadh 11451, Saudi Arabia
[2] King Abdullah Institute for Nanotechnology, King Saud University, Riyadh 11451, Saudi Arabia; shaykha.alzahly@hotmail.com
[3] Nuclear Sciences Research Institute, King Abdulaziz City for Science and Technology, Riyadh 11442, Saudi Arabia; almoiqli@kacst.edu.sa
* Correspondence: mrahaman@ksu.edu.sa (M.R.); aaldalbahi@ksu.edu.sa (A.A.); Tel.: +966-114-675-883 (M.R. & A.A.)

S1: Solubility of Ppy-IC Composite

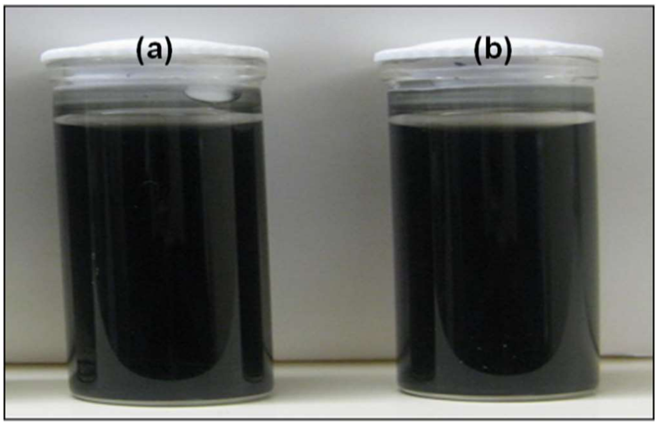

Figure S1. Images showing the solubility of PPy-IC composite solutions in: (**a**) Milli-Q water and (**b**) DMSO.

S2: Photographs of Ppy-Carrageenan Film

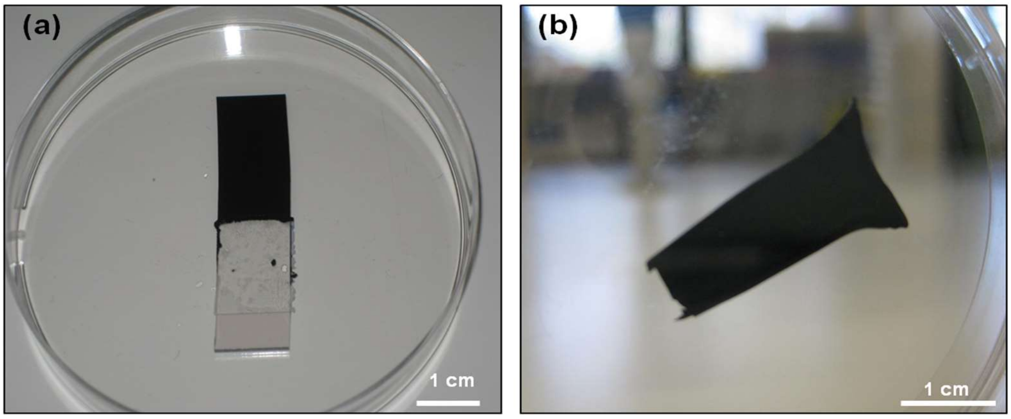

Figure S2. Photographs of (**a**) PPy-carrageenan film deposited onto ITO glass after the polymerization and (**b**) the film after peeling off from the ITO glass.

S3: Free-Standing Films Prepared by Evaporative Casting

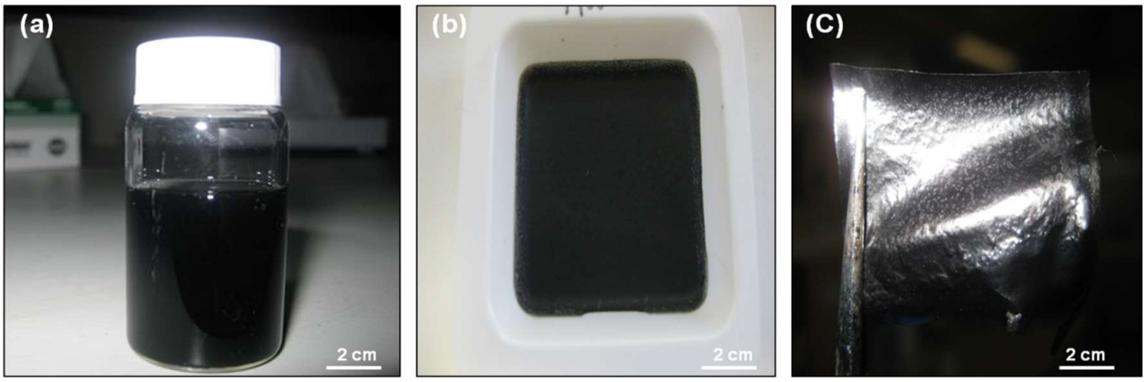

Figure S3. Photographs of (**a**) PPy-IC composite solution, (**b**) PPy-IC composite solution during the evaporation into the plastic substrate and (**c**) PPy-IC composite film after peeling off from the plastic substrate.

© 2018 by the authors. Submitted for possible open access publication under the terms and conditions of the Creative Commons Attribution (CC BY) license (http://creativecommons.org/licenses/by/4.0/).
